# Overt and Covert Effects of Mental Fatigue on Attention Networks: Evidence from Event-Related Potentials during the Attention Network Test

**DOI:** 10.3390/brainsci14080803

**Published:** 2024-08-10

**Authors:** Caterina Pauletti, Daniela Mannarelli, Francesco Fattapposta

**Affiliations:** Department of Human Neurosciences, Sapienza University of Rome, Viale dell’Università 30, 00185 Rome, Italy; daniela.mannarelli@uniroma1.it (D.M.); francesco.fattapposta@uniroma1.it (F.F.)

**Keywords:** attention, ERPS, ANT, mental fatigue, alerting, orienting, executive, conflict, P3, N2

## Abstract

Mental fatigue is a variation in the psychophysiological state that subjects encounter during or after prolonged cognitive activity periods, affecting top-down attention and cognitive control. The present study aimed to investigate the effects of mental fatigue on attention in the context of the three attention networks according to the Posnerian model (alerting, orienting, and executive networks) by combining the Attentional Network Test (ANT) and event-related potentials technique. Thirty healthy subjects were enrolled in the study. A continuous arithmetic task lasting one hour induced mental fatigue, and EEG recordings were conducted before and after the task while subjects were performing the ANT. The efficiencies of three networks were comparable between groups, while RTs shortened only in the control group and the accuracy related to the alerting and conflict networks declined only after mental effort. Mental fatigue reduced N1 amplitude during alerting network engagement and p3 amplitude during orienting. It also reduced N2 and P3 amplitude during the conflict, particularly the incongruent target-locked response. These findings underscore the covert effects of mental fatigue on attention, suggesting that even in healthy young subjects, compensatory mechanisms may maintain adequate overt performances, but fatigue still has a detrimental effect on top-down attentional mechanisms.

## 1. Introduction

Attention is a major and complex cognitive function defined as correctly allocating processing resources to relevant stimuli [1]. It can be voluntary or automatically directed to a selective location or task, prioritizing its focus and filtering out other stimuli (selective attention); it can be kept on it for a prolonged period of time (sustained attention), or it can be switched and divided between stimuli (divided attention) [2,3]. One early influential model, based on the hypothesis that attention has various neural underpinnings, was proposed by Michael Posner; the Attention Network Theory stated that there are at least three components of attention, i.e., alerting, orienting, and executive [4,5,6]. Briefly, alerting prepares the system for stimuli to enable responsiveness; orienting directs attention to targets; and executive (or conflict) monitors and solves conflicts between competing information [5,6]. These three attentional processes are subserved by functionally independent and anatomically distinct networks predominantly modulated by noradrenergic, cholinergic, and dopaminergic neurotransmitter systems, respectively, and can be easily activated in experimental settings with a short task first proposed by Fan, combining the cued reaction time task and the flanker task, called the Attentional Network Test (ANT) [7]. Neuroimaging studies provided broad evidence of the activation of brain areas such as the thalamus and the right frontal and parietal cortices, which are part of the ventral attention system, related to alerting; frontal eye fields and the superior parietal lobe related to orienting (regions that are part of the dorsal attention system); and the anterior cingular cortex (ACC) and the lateral prefrontal cortex (part of the salience network) related to conflict [3,8].

Besides neuroimaging, a reliable tool to explore the neural processes underlying the activation of attentional networks with exceptional temporal resolution is represented by the event-related potentials (ERPs) [9]. In particular, the modulation of ERPs during the ANT has been well documented on the N1, N2, and P3 components [10]. The N1 component of ERPs is an early positive deflection related to selective attention and visual discrimination [11]. During the ANT, an enhancement in the amplitude of the cue-locked target N1 component was documented both for alerting and orienting network activation, indicating that N1 is modulated by attention focused on the attended location of the target [10,11]. The P3 is a late positive component that peaks about 300 ms after a behaviorally relevant stimulus and is elicited by paradigms involving target detection [12]. Its amplitude is related to a number of attentive resources for the task relevance and stimulus probability, and its latency reflects the stimulus evaluation time [11,13]. A modulation of P3 amplitude (frontal P3 increment and parietal P3 decrement) was observed during the engagement of the conflict network [10]. In addition to P3, another component of ERPs has been described, which is related to cognitive control in conflict resolution and response inhibition; N2, which is a negative peak in the averaged ERP’s waveform (the second negative peak, following the first prominent frontocentral one around 100 ms for auditory and around 180 ms for visual modality), proved to be larger following no-go stimuli in which the response selection in the conflicting situation is highly stressed [14].

Attention networks could be influenced at many levels by several physiological and pathological factors; among them, mental fatigue is a variation in the psychophysiological state that subjects encounter during or after prolonged cognitive activity periods that require work efficiency [15,16], and a common phenomenon in this epoch, related to multiple causes, such as prolonged working hours, inadequate sleep, and loss of willingness [17], has been extensively associated with increased errors, a transitory worsening of response readiness, and a decline in attentional functioning [16,18,19]. Several studies conducted on healthy subjects with ERPs have demonstrated that mental fatigue may lead to reduced action monitoring [20], attenuated resource allocation and error monitoring [21], a diminished influence of advanced information on stimulus processing [16], and impaired preattentive processing [22].

Moreover, fatigue is an important symptom in many chronic diseases and neurological disorders [23]; therefore, the effects of fatigue on attention have been investigated in a clinical population. For example, studies on chronic fatigue syndrome demonstrated that patients, regardless of the presence of depression, presented with longer latencies than controls in the conflict condition, especially in response to the incongruent target [24,25,26]. The delayed processing of conflict information was associated with the presence of attentional bias towards health-threat stimuli, indicating that in this syndrome dominated by fatigue, unlike in patients with functional neurological disorders, worse executive control could lead to an inability to rule out salient but task-irrelevant stimuli [25,26]. Executive control, which subserves many cognitive operations such as planning, response inhibition, working memory, and problem-solving, is a crucial function for goal-directed behavior and requires information processing to be carried out in an adequate time, in order to efficiently switch between tasks [27]. Task switching physiologically increases response speed (“switch cost”) and mental fatigue could possibly enhance this cost. These data point to a prominent influence of fatigue on top-down attention and cognitive control, strictly related to the frontal basal ganglia circuitry [28], as also demonstrated by data on Parkinson’s disease, a neurodegenerative disorder characterized by an overall dysfunction of the basal ganglia circuitry (including the striato-thalamus-prefrontal circuit), produced by dopamine deafferentation in the striate nucleus; parkinsonian patients with fatigue displayed worse conflict efficiency at the ANT than parkinsonian patients without fatigue and controls [29].

The present study aimed to disentangle mental fatigue’s effects on attention, specifically in the context of the three attentional networks using behavioral (RT) and psychophysiological (ERPs) measures to detect variations in overt and covert responses induced by mental fatigue. Mental fatigue was provoked by a continuous arithmetic task lasting 1 h, and the EEG recordings were conducted before and after the task while subjects were performing the ANT, avoiding the time-on-task effect. In fact, most cited studies have utilized the time-on-task effect on behavioral and psychophysiological measures to exert mental fatigue. However, interpretations of changes in performance and in ERP parameters, especially amplitude, with time-on-task as correlates of mental fatigue must be treated with caution; the involvement in a cognitive task for a longer period may also lead, beyond mental fatigue, to learning and adaptation effects and to changes in the motivation to continue with the task [30].

Given the current definition of mental fatigue and the theoretical model implicating the prefrontal basal circuitry as a core substrate of fatigue, we here hypothesized that after becoming mentally fatigued, subjects would experience difficulties mainly related to the executive network, with relative sparing of the alerting and the orienting networks. Moreover, we expected that the psychophysiological measures related to the executive network, meaning the N2 and the P3 elicited by incongruent stimuli that require, by definition, more cognitive control and conflict resolution to exert a correct response, would be more influenced by mental fatigue.

## 2. Materials and Methods

### 2.1. Subjects and Methods

#### 2.1.1. Subjects

Thirty healthy right-handed subjects, fifteen in the fatigue group (F) (ten females, mean age 29.3 ± 5.4 years) and fifteen in the control group (NF) (twelve females, mean age 28.5 ± 4.7 years), were enrolled in our study. All participants were free from any history of neurological or psychiatric disease, head injury, or excessive alcohol consumption and were not taking any medication with central nervous effects. Prior to the experiment, all subjects signed an informed consent form. The study, with protocol number 1965/15, was approved by the Local Medical Ethics Committee (Sapienza University of Rome) and conducted in full compliance with the Declaration of Helsinki, ensuring the highest ethical standards were upheld.

#### 2.1.2. Procedure

**Figure 1:**
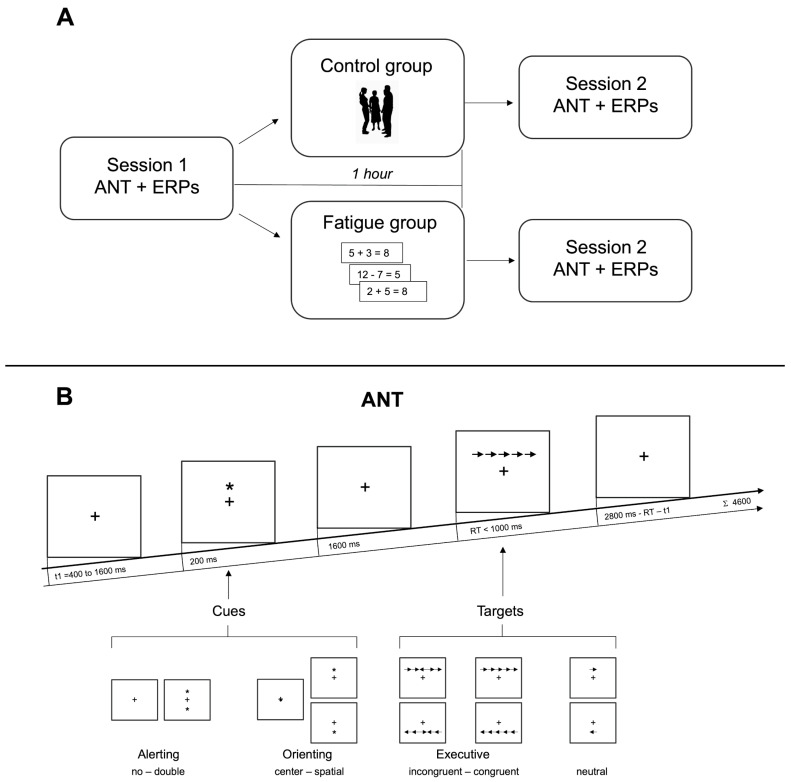
(**A**): Graphic drawing representing the entire procedure. (**B**): An example of the ANT task with the time course. +: fixation point; *: cue.

The experiment was a comprehensive process consisting of the ANT performed at two-time points, each separated by a one-hour interval. During the ANT, the EEG signals were recorded from the scalp, a crucial step in our data collection process (see Figure 1).

Subjects were randomly assigned to two groups (www.random.org, accessed on 6 July 2015): the fatigue group (F), which performed 1 h of continuous arithmetic tasks, and the control group (NF), which performed 1 h of leisure activity. They were instructed to abstain from alcohol and caffeine 24 h before the experiment.

Prior to each recording session, subjects were asked to indicate on a rating scale varying from 0 (not at all) to 10 (maximal) the degree of aversion to performing the incoming task and to complete the profile of mood states (POMS—only the fatigue subscale was calculated [31].

During the 1 h interval, the F group performed a continuous arithmetic task consisting of a sequence of equations (four single digits, three operators with only additions and subtractions allowed, and the target sum) displayed on a monitor, with an inter-trial interval of 1 s. Subjects were asked to report whether the equation was true or false on a schedule. The NF group was instead instructed to read a magazine, take a little walk, or chat among themselves during the 1 h interval.

#### 2.1.3. EEG Recording

Participants were seated in an anatomic chair in a partially soundproof, faradized, and light-attenuated room. The electrophysiological signals were recorded using a 21-channel cap. Active electrodes were at the F3, Fz, F4, C3, Cz, C4, P3, Pz, and P4 sites, according to the International 10–20 System, referred to as linked mastoids and grounded at the forehead. A vertical electro-oculogram (V-EOG) was recorded above and below the left eye. A horizontal EOG (H-EOG) was also achieved with electrodes at the two external canthi. All inter-electrode impedances were kept below 3 KOhm. EEG and EOG signals were filtered using a 0.01–30 Hz bandpass. A notch filter was also applied. The data were digitized with an analog/digital (A/D) converter at a sampling rate of 1024 Hz and stored on a hard disk. A Mizar Sirius EEG-EP multifunctional system was used.

#### 2.1.4. Paradigm

The ANT we adopted was the one first proposed by Fan et al. [7]. Adjustments were made to elicit the psychophysiological components.

The task combines cue detection with a flanker task. Cues are represented by asterisks and targets by arrows. There are four cue conditions: (1) “no cue” (NC): the target is not preceded by an asterisk; (2) “double cue” (DC): the target is preceded by an asterisk appearing both above and below the center of the screen at the same time; (3) “center cue” (CC): the target is preceded by an asterisk appearing at the center of the screen; (4) “spatial cue” (SC): the asterisk appears either above or below the center, indicating the exact location of the target. There are three target conditions: (1) “neutral”: flankers do not surround the target; (2) “congruent” (C): the central target arrow is surrounded by two arrows on each side all pointing in the same direction; (3) “incongruent” (I): the two pairs of arrows surrounding the central target point in the opposite direction. Participants are asked to respond as fast and accurately as possible by pressing one button for the left-pointing arrow and another for the right-pointing arrow (see Figure 1). Measuring how RT is influenced by alerting cues (NC versus DC), spatial cues (CC versus SC), and flankers (C versus I) provides a measure of the efficiency of the three attentional networks. Indeed, the DC keeps attention towards to two potential target locations (below or above the fixation point), and it also alerts the subject to the impending arrival of the target stimulus, recruiting the alerting network. Both CC and SC are alerting cues, but only the SC provides spatial information that allows subjects to start orienting attention to the appropriate location while attending to the target. It therefore activates the alerting and the orienting network. Moreover, because of the conflicting information carried by the I flankers, more effort is required in the executive network while responding to this type of target when compared to the C flanker’s target.

In our experiment, the test consisted of a 24-trial practice block, during which subjects received feedback on their accuracy, and two experimental blocks without feedback. Each experimental block consists of 96 trials (two repetitions of 48 conditions: 4 cue types × 2 target locations (above/below) × 2 target directions (right/left) × 3 flanker conditions).

Each trial was composed of a first fixation that varies randomly between 400 and 1600 ms. Next, the cue was presented for 200 ms (or, in the NC condition, a second fixation lasting 200 ms). The interval between the cue and target stimulus was modified with respect to the original task by prolonging it from 400 ms to 1600 ms. The target and flankers, presented simultaneously, lasted until the subject responded or for 1000 ms if there was no response. The last post-target fixation period depended on the reaction time and the first fixation (it lasts max 2800 ms minus the reaction time (RT) minus the duration of the first fixation) (see Figure 1).

#### 2.1.5. ERP Analysis

EEG data were clipped offline into epochs of 800 ms with a baseline correction of 100 ms before each stimulus. A first automatic procedure was used to reject trials containing drift deflection more than ±100 μV in any channel including EOG, as recommended by Duncan et al. in their guidelines for eliciting, recording, and quantifying ERPs [9]. A further selection was performed in the offline analysis to reject ocular artifacts (eye movements/blinks) according to the step-function algorithm described by Luck 2014 [11] and that was implemented in our analyzer software (ERPLAB Toolbook, v6.1.4). Specifically, trials containing artifacts were eliminated by computing the cross-covariance between the single-trial EOG waveform and a 200 ms step function and rejecting trials in which the maximum covariance exceeded a ±15-μV threshold. Lastly, artifact detection was verified by visual inspection. Artifact rejection accounted for 36.2 ± 21.9/112 (32.3%) of the trials for each subject per session.

The ERPs for each subject for the two ANT sessions were evaluated. All artifact-free trials were averaged per cue (no cue, double cue, spatial cue, center cue) and target stimulus (congruent and incongruent) and were filtered with a low-pass digital filter of 20 Hz. The mean number of trials included was 71 ± 19.9/112 (63.4%) for the F group (*cue*: 29.5 ± 8.3/48 (61.5%):—29.2 ± 8.3 for Session 1, 29.8 ± 8.5 for Session 2; *target*: 41.5 ± 11.5/64 (64.8%)—39 ± 9.7 for Session 1, 44 ± 12.7 for Session 2) and 80.5 ± 23.1/112 (71.9%) for the NF group (*cue*: 32.8 ± 10.1/48 (68%)—33.2 ± 9.7 for Session 1, 32.3 ± 10.7 for Session 2; *target*: 47.8 ± 13/64 (75%)—47.1 ± 12.6 for Session 1, 48.5 ± 13.7 for Session 2).

Scalp electrode activity was evaluated at all electrode sites where Fz, Cz, and Pz were measured. Fz, Cz, and Pz were chosen for measures because the ERP responses were the largest in the midline locations. The components’ amplitude was identified by means of baseline-to-peak measurements [32].

The N1 component was identified as the most negative peak between 140 and 280 ms after the target stimulus. Given the fact that its amplitude is modulated by the engagement of the alerting and the orienting networks, N1 amplitude was used as a marker of covert response to the mental fatigue’s influence on these attention networks, and it was analyzed in conformity with the behavioral analysis, meaning the following:

- Alerting: target N1_nocue_ vs. target N1_doublecue_;
- Orienting: target N1_centralcue_ vs. target N1_spatialcue_;

The cue trials were collapsed over all target conditions.

The N2 component was identified as the most negative peak between 240 and 340 ms after target stimulus.

Given the fact that N2 is modulated by the cognitive control to incorrect response preparation, its amplitude was used as a marker of covert response to mental fatigue’s influence on the conflict network, and it was analyzed in conformity with the behavioral analysis, meaning the following:

- Conflict: target N2_incongruent_ vs. target N2_congruent_;

The target trials collapsed over all cue conditions.

The P3 component was identified as the largest positive deflection between 350 and 700 ms after the target stimulus. This component is related to selective attention and stimulus discrimination. Its amplitude was, therefore, used as a marker of the effects exerted by mental fatigue on the three attentional networks.

The P3s locked to the target were analyzed in conformity with the behavioral analysis, meaning the following:

- Alerting: target P3_nocue_ vs. target P3_doublecue_;
- Orienting: target P3_centralcue_ vs. target P3_spatialcue_;

The cue trials collapsed over all target conditions.

- Conflict: target P3_incongruent_ vs. target P3_congruent_;

The target trials collapsed over all cue conditions.

### 2.2. Statistical Analyses

Data are expressed as the mean (±1 standard deviation) for continuous variables and proportions for categorical variables. The Kolmogorov–Smirnov test was applied to assess the data’s normal distribution and ensure that the assumption of normality was not violated for any of the data (*p* > 0.05).

#### 2.2.1. Overt Responses: ANT Analysis

Outcome measures for the ANT are the three networks’ efficiencies, RT and accuracy.

For the calculation of RT, an initial data reduction led to the exclusion of trials with incorrect responses, trials with an RT < 200 or >1200 ms, and trials with an RT more than the subject’s mean RT ± 2SD.

In line with Fan [7], a subtraction method was employed to isolate the efficiency of the three attentional networks. Specifically, the alerting network efficiency was indexed by subtracting the mean RT on DC trials from the mean RT on NC trials; the orienting network efficiency was indexed by subtracting the mean RT on SC trials from the mean RT on CC trials; and the executive network efficiency was indexed by subtracting the mean RT on C target trials from the mean RT on I target trials. Higher subtraction scores for the alerting and orienting effects indicate greater efficiency, while a lower subtraction score for the executive network signifies higher efficiency.

Accuracy is defined as the proportion of correct responses based on all the trials and as the absolute number of errors.

The efficiency of the alerting, orienting, and executive networks was analyzed separately using a mixed-model rmANOVA, with “timing” (pre, post) as the within-subject factor and the “group” (F, NF) as the between-subject factor.

The RT and the absolute number of errors were analyzed using a mixed-model rmANOVA with the “group” (F, NF) as the between-subject factor and with the timing (pre, post) as the within-subject factor. Moreover, depending on the network under study, one more within-subject factor was added: for the alerting network, the “cue” (NC, DC); for the orienting network, the “cue” (CC, SC); and for the conflict network, the “target” (C, I).

A post hoc correction, according to Bonferroni, was applied when required. Degrees of freedom were adjusted, when necessary, using the Greenhouse–Geisser epsilon coefficient for possible violations of the sphericity assumption, and corrected *p* values were reported; the original degrees of freedom were reported together with their correction factor epsilon. A *p* < 0.05 was considered statistically significant.

Regarding accuracy, data were not normally distributed, probably because of the ceiling effect; thus, no further analyses were conducted.

#### 2.2.2. Covert Responses: ERP Analyses

For the alerting network, the cue-locked target N1 and cue-locked target P3 latencies and amplitudes were analyzed separately by means of mixed-model rmANOVA, with the “group” (F, NF) as the between-subject factor and the “electrode” (Fz, Cz, Pz), the timing (pre, post), and the “cue” (NC, DC) as within-subject factors.

For the orienting network, the cue-locked target N1 and cue-locked target P3 latencies and amplitudes were analyzed separately by means of mixed-model rmANOVA, with the “group” (F, NF) as the between-subject factor and the “electrode” (Fz, Cz, Pz), the timing (pre, post), and the “cue” (CC, SC) as within-subject factors.

For the executive network, the N2 and P3 latencies and amplitudes were analyzed separately by means of mixed-model rmANOVA with the “group” (F, NF) as the between-subject factor and the “electrode” (Fz, Cz, Pz), the timing (pre, post), and the “target” (C, I) as within-subject factors.

According to Krats et al. [33], this allowed for the display of ANT network effects modulated by fatigue at the ERP level.

A post hoc correction, according to Bonferroni, was applied when necessary. When necessary, degrees of freedom were adjusted using the Greenhouse–Geisser epsilon coefficient for possible violations of the sphericity assumption, and corrected *p* values were reported; the original degrees of freedom were reported together with their correction factor epsilon. A *p* < 0.05 was considered statistically significant.

The analyses were performed using the SPSS statistical package (Version 25.0).

## 3. Results

All the subjects completed the task. The subjects’ self-reported ratings of aversion to the task and fatigue were significantly different prior to and after the sessions in the F group alone (Table 1). The mean RT and accuracy values are shown in Table 2.

### 3.1. Overt Responses

#### 3.1.1. Network Efficiency

As regards the alerting network efficiency, the ANOVA did not reveal a main effect of the “group” factor (F_(1,28)_ = 0.22, *p* = 0.64, η^2^_p_ = 0.08). It did not reveal a main effect of the “timing” factor (F_(1,28)_ = 0.53, *p* = 0.47, η^2^_p_ = 0.018). The “group” × “timing” interaction was not significant (F_(1,28)_ = 0.60, *p* = 0.44, η^2^_p_ = 0.021).

As regards the orienting network efficiency, the ANOVA revealed a main effect of the “group” factor (F_(1,28)_ = 5.57, *p* = 0.03, η^2^_p_ = 0.16). It did not reveal a main effect of the “timing” factor (F_(1,28)_ = 0.05, *p* = 0.94, η^2^_p_ < 0.001). The “group” × “timing” interaction was not significant (F_(1,28)_ = 0.37, *p* = 0.55, η^2^_p_ = 0.013).

**Figure 2:**
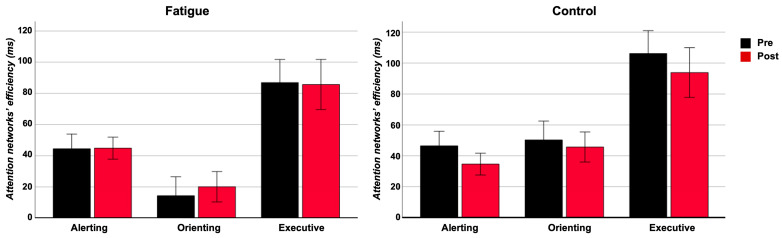
Attention networks’ efficiency for fatigue and control groups for the two sessions. Error bars indicate ±1 SE.

As regards the executive network efficiency, the ANOVA did not reveal a main effect of the “group” factor (F_(1,28)_ = 0.47, *p* = 0.49, η^2^_p_ = 0.017). It did not reveal a main effect of the “timing” factor (F_(1,28)_ = 0.57, *p* = 0.46, η^2^_p_ = 0.02). The “group” × “timing” interaction was not significant (F_(1,28)_ = 0.38, *p* = 0.54, η^2^_p_ = 0.013) (see Figure 2).

#### 3.1.2. RT

As regards the alerting, the ANOVA did not reveal a main effect of the “group” factor (F_(1,28)_ = 0.75, *p* = 0.39, η^2^_p_ = 0.03). It revealed a main effect of the “cue” factor (F_(1,28)_ = 104.9, *p* < 0.001, η^2^_p_ = 0.79), with a significantly shorter RT after DC. The “group” × “timing” interaction was significant (F_(1,28)_ = 17.3, *p* < 0.001, η^2^_p_ = 0.38). After Bonferroni’s correction, the RT was significantly reduced in the NF group alone (pre: 577.7, post: 536.3; *p* < 0.001), while a tendency of a prolongation in RT was present in the F group (pre: 571.7, post: 590.9; *p* = 0.07).

As regards the orienting, the ANOVA did not reveal a main effect of the “group” factor (F_(1,28)_ = 1.35, *p* = 0.25, η^2^_p_ = 0.05). It revealed a main effect of the “cue” factor (F_(1,28)_ = 35.4, *p* < 0.001, η^2^_p_ = 0.56), with a significantly shorter RT after SC. The “group” × “timing” interaction was significant (F_(1,28)_ = 15.0, *p* = 0.001, η^2^_p_ = 0.35). After Bonferroni’s correction, the RT was significantly reduced in the NF group alone (pre: 542.1, post: 498.9; *p* < 0.001), while in the F group, the RT remained stable (pre: 543.7, post: 559.3; *p* = 0.16).

**Figure 3:**
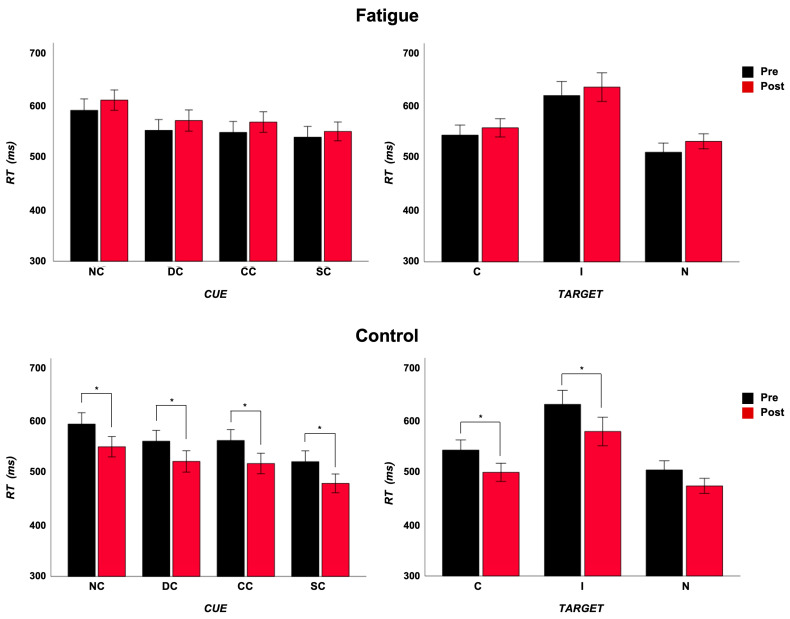
RTs in fatigue and control groups collapsed for cues and targets in the two sessions. Error bars indicate ±1 SE. * *p* < 0.05.

As regards the conflict, the ANOVA did not reveal a main effect of the “group” factor (F_(1,28)_ = 0.74, *p* = 0.40, η^2^_p_ = 0.03). It revealed a main effect of the “target” factor (F_(1,28)_ = 82.3, *p* < 0.001, η^2^_p_ = 0.38), with a significantly longer RT after I target, and of the “timing” factor (F_(1,28)_ = 4.65, *p* = 0.04, η^2^_p_ = 0.14), with a shorter RT post manipulation. The “group” × “timing” interaction was significant (F_(1,28)_ = 17.4, *p* < 0.001, η^2^_p_ = 0.38). After Bonferroni’s correction, the RT was significantly reduced in the NF group alone (pre: 587.3, post: 539.7; *p* < 0.001), while in the F group, the RT remained stable (pre: 582.3, post: 597.4; *p* = 0.16) (Figure 3).

#### 3.1.3. Accuracy

As regards the alerting, the ANOVA did not reveal a main effect of the “group” factor (F_(1,28)_ = 2.25, *p* = 0.14, η^2^_p_ = 0.67), of the “cue” factor (F_(1,28)_ = 2.5, *p* = 0.12, η^2^_p_ = 0.08), or of the “timing” factor (F_(1,28)_ = 1.2, *p* = 0.27, η^2^_p_ = 0.04). The “group” × “timing” interaction was significant (F_(1,28)_ = 27.4, *p* < 0.001, η^2^_p_ = 0.49). After Bonferroni’s correction, errors were significantly lower after manipulation in the NF group (pre: 0.9, post: 0.5; *p* = 0.007); they were significantly higher after manipulation in the F group (pre: 0.7, post: 1.4; *p* < 0.001).

As regards the orienting, the ANOVA did not reveal a main effect of the “group” factor (F_(1,28)_ = 0.04, *p* = 0.85, η^2^_p_ = 0.001), of the “cue” factor (F_(1,28)_ = 1.5, *p* = 0.22, η^2^_p_ = 0.05), or of the “timing” factor (F_(1,28)_ = 0.12, *p* = 0.73, η^2^_p_ = 0.004). No significant interactions emerged.

**Figure 4:**
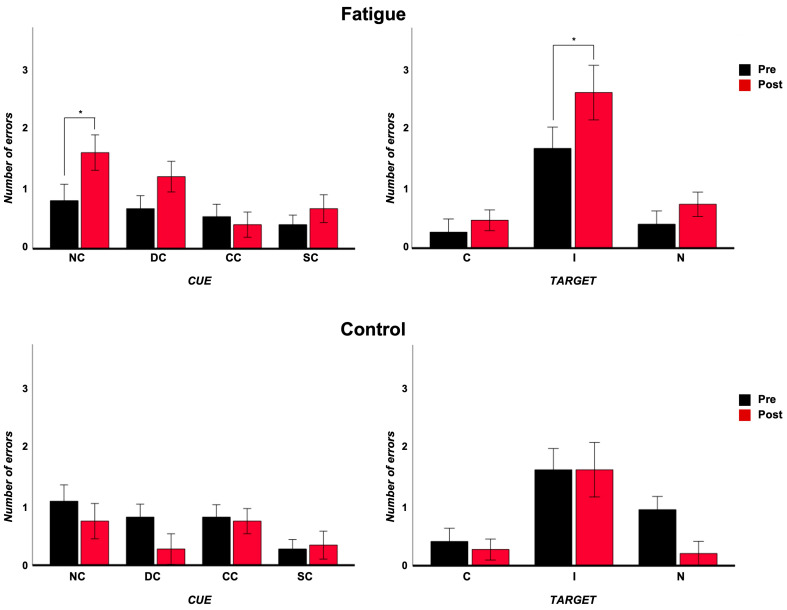
Number of errors in fatigue and control groups collapsed for cues and targets in the two sessions. Error bars indicate ±1 SE. * *p* < 0.05.

As regards the conflict, the ANOVA did not reveal a main effect of the “group” factor (F_(1,28)_ = 0.90, *p* = 0.35, η^2^_p_ = 0.03). It revealed a main effect of the “target” factor (F_(1,28)_ = 29.3, *p* ≤ 0.001, η^2^_p_ = 0.51), with a significantly higher number of errors after I target, and of the “timing” factor (F_(1,28)_ = 4,08, *p* = 0.05, η^2^_p_ = 0.13), with a higher number of errors post manipulation. The “group” × “timing” interaction was significant (F_(1,28)_ = 6.5, *p* = 0.16, η^2^_p_ = 0.19). After Bonferroni’s correction, errors were significantly higher after manipulation in the F group (pre: 0.97, post: 1.5; *p* = 0.03), while they were comparable in the NF group (pre: 1, post: 0.9; *p* = 0.70) (Figure 4).

### 3.2. Covert Responses: ERP Results

#### 3.2.1. Alerting—N1

As regards the amplitude, the ANOVA did not reveal a main effect of the “group” factor (F_(1,28)_ = 1.38, *p* = 0.24, η^2^_p_ = 0.05). It revealed a main effect of the “cue” factor (F_(1,28)_ = 5.06, *p* = 0.03, η^2^_p_ = 0.15), with a higher amplitude for the DC, and the “electrode” factor (F_(2,56)_ = 4.07, *p* = 0.02, η^2^_p_ = 0.13), with a higher amplitude in parietal sites; it did not reveal a main effect of the “timing” factor (F_(1,28)_ = 1.25, *p* = 0.27, η^2^_p_ = 0.04).

**Figure 5:**
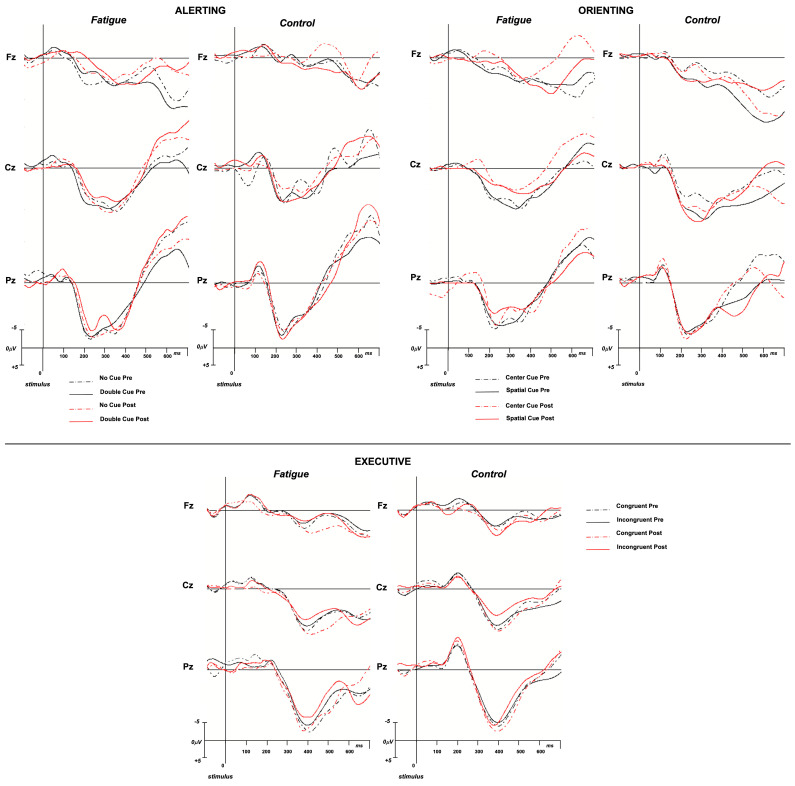
ERP traces in mid-line scalp locations for alerting, orienting, and executive networks, both for fatigue and control groups.

The “group” × “timing” interaction was significant (F_(1,28)_ = 4.09, *p* = 0.05, η^2^_p_ = 0.13). After Bonferroni’s correction, the N1 amplitude was significantly reduced in the F group alone (pre: −3.6, post: −2.9; *p* = 0.034), while no difference emerged in the NF group (pre: −3.7, post: −3.8; *p* = 0.53). No significant difference emerged between groups as regards latency (Figure 5).

#### 3.2.2. Alerting—P3

As regards the amplitude, the ANOVA did not reveal a main effect of the “group” factor (F_(1,28)_ = 0.81, *p* = 0.38, η^2^_p_ = 0.03). It revealed a main effect of the “cue” factor (F_(1,28)_ = 8.9, *p* = 0.006, η^2^_p_ = 0.24), with a higher amplitude for the DC, and the “electrode” factor (F_(2,56)_ = 16.4, *p* < 0.001, η^2^_p_ = 0.37), with a higher amplitude in parietal sites; it did not reveal a main effect of the “timing” factor (F_(1,28)_ = 0.77, *p* = 0.39, η^2^_p_ = 0.03).

The “group” × “timing” interaction was not significant (F_(1,28)_ = 2.02, *p* = 0.17, η^2^_p_ = 0.07). No significant difference emerged between groups regarding latency (Figure 5).

#### 3.2.3. Orienting—N1

As regards the amplitude, the ANOVA did not reveal a main effect of the “group” factor (F_(1,28)_ = 0.07, *p* = 0.31, η^2^_p_ = 0.04). It revealed a main effect of the “electrode” factor (F_(2,56)_ = 3.71, *p* = 0.03, η^2^_p_ = 0.12), with a higher amplitude in parietal sites; it did not reveal a main effect of the “cue” factor (F_(1,28)_ = 0.06, *p* = 0.80, η^2^_p_ = 0.002) or of the “timing” factor (F_(1,28)_ = 2.77, *p* = 0.11, η^2^_p_ = 0.09). The “group” × “timing” interaction was not significant (F_(1,28)_ = 1.87, *p* = 0.18, η^2^_p_ = 0.06). No significant difference emerged between groups regarding latency (Figure 5).

#### 3.2.4. Orienting—P3

As regards the amplitude, the ANOVA did not reveal a main effect of the “group” factor (F_(1,28)_ = 3.03, *p* = 0.09, η^2^_p_ = 0.10). It revealed a main effect of the “electrode” factor (F_(2,56)_ = 21.4, *p* < 0.001, η^2^_p_ = 0.43), with higher amplitude in parietal sites. It did not reveal a main effect of the “cue” factor (F_(1,28)_ = 0.87, *p* = 0.36, η^2^_p_ = 0.03) and the “timing” factor (F_(1,28)_ = 1.88, *p* = 0.18, η^2^_p_ = 0.06).

The “group” × “timing” interaction was significant (F_(1,28)_ = 4.4, *p* = 0.04, η^2^_p_ = 0.14). After Bonferroni’s correction, the P3 amplitude was significantly reduced in the F group alone (pre: 7.0, post: 5.4; *p* = 0.02), while no difference emerged in the NF group (pre: 7.3, post: 7.6; *p* = 0.6). No significant difference emerged between groups regarding latency (Figure 5).

#### 3.2.5. Conflict—N2

As regards the amplitude, the ANOVA did not reveal a main effect of the “group” factor (F_(1,28)_ = 8.7, *p* = 0.70, η^2^_p_ = 0.05). It revealed a main effect of the “target” factor (F_(1,28)_ = 3.7, *p* = 0.06, η^2^_p_ = 0.12), with a higher amplitude for the I target; the “electrode” factor (F_(2,56)_ = 5.98, *p* = 0.004, η^2^_p_ = 0.18), with a higher amplitude in frontal sites; and the “timing” factor (F_(1,28)_ = 5.36, *p* = 0.03, η^2^_p_ = 0.16) with a higher amplitude pre-manipulation.

The “group” × “timing” interaction was significant (F_(1,28)_ = 9.4, *p* = 0.005, η^2^_p_ = 0.25); after Bonferroni’s correction, the N2 amplitude was significantly reduced in the F group alone (pre: −2.4, post: −0.31; *p* = 0.01), while no difference emerged in the NF group (pre: −1.6, post: −1.9; *p* = 0.53).

Moreover, the “group” × “timing” × “target” interaction was significant (F_(1,28)_ = 3.1, *p* = 0.05, η^2^_p_ = 0.10); after Bonferroni’s correction, the N2 amplitude was significantly reduced in the F group only for the I target (pre: −3.3, post: −0.08; *p* < 0.001), while no difference emerged for the C target (pre: −1.6, post: −0.7; *p* = 0.28).

No significant difference emerged between groups regarding latency (Figure 5).

#### 3.2.6. Conflict—P3

As regards the amplitude, the ANOVA did not reveal a main effect of the “group” factor (F_(1,28)_ = 1.2, *p* = 0.27, η^2^_p_ = 0.04). It revealed a main effect of the “target” factor (F_(1,28)_ = 8.6, *p* = 0.007, η^2^_p_ = 0.23), with a higher amplitude for the C target, and the “electrode” factor (F_(2,56)_ = 56.7, *p* < 0.001, η^2^_p_ = 0.67), with a higher amplitude in parietal sites. No main effect of the “timing” factor emerged (F_(1,28)_ = 2.9, *p* = 0.10, η^2^_p_ = 0.09).

The “group” × “timing” interaction was significant (F_(1,28)_ = 13.0, *p* = 0.001, η^2^_p_ = 0.32); after Bonferroni’s correction, the P3 amplitude was significantly reduced in the F group alone (pre: 7.1, post: 5.6; *p* = 0.001), while no difference emerged in the NF group (pre: 6.3, post: 7.4; *p* = 0.18).

Moreover, the “timing” × “target” interaction was significant (F_(1,28)_ = 12.7, *p* = 0.001, η^2^_p_ = 0.31); after Bonferroni’s correction, the P3 amplitude was significantly reduced for the I target (pre: 6.6, post: 5.2; *p* = 0.001), while no difference emerged for the C target (pre: 7.4, post: 7.7; *p* = 0.35).

No significant difference emerged between groups regarding the P3 latency (Figure 5).

## 4. Discussion

The present study aimed to study mental fatigue’s overt and covert effects on attention networks induced by one hour of exhausting mental activity, which differed from those engaged in the psychophysiological task.

The first observation from our data is that mental fatigue does not influence attention networks’ efficiency. Attention network efficiency is derived from how the RT is influenced by the characteristics of cues and flankers, specifically by alerting cues (no cue versus double cue), spatial cues (center cue versus spatial cue), and flankers (congruent versus incongruent). In detail, the alerting network efficiency is operationally defined in terms of how much visual cues that carry temporal information reduce the RT with respect to the absence of cues, the orienting network efficiency in terms of how much spatial information reduces the RT with respect to alerting information alone, and the conflict network efficiency in terms of how much conflicting information delays the RT with respect to congruent information [7].

Consequently, this behavioral measure provides data regarding the ability to process information carried out by stimuli correctly and to efficiently use them to reduce RT (which is one of the main task’s demands, above accuracy). However, it does not provide data regarding the processing speed or the number of resources engaged in the task. In line with these data, a recent study demonstrated that no temporal decrement was present in the ANT indices for the efficiency of the three attention networks, both after 60 min of ANT performance and after manipulations of trial blocking and stimulus degradation aimed to increase resource depletion, indicating that attention networks are quite resilient to prolonged activation [34]. As regards the alerting and the conflict networks, their efficiency came at the expense of accuracy: subjects that experienced mental fatigue were able to engage these networks but made more errors.

An interesting pattern of behavioral performance emerged when analyzing RTs, in addition to their operational utility in defining network efficiency. Independently from the cue–flanker combination, in the NF group, RTs significantly decreased in the second session. On the contrary, the RT in the F group remained stable, indicating that mental fatigue prevented the shortening of RTs due to task repetition and inhibited the physiological learning process. This observation aligns with a recent experiment by Khojasteh Moghani et al. [35] that demonstrated that mental fatigue interferes with motor learning, especially when the feedback is self-controlled.

When analyzing the covert responses provided by ERPs, much more interesting data emerged. First, as regards the alerting network, the N1 amplitude was reduced after one hour of exhausting mental activity, while no differences emerged in the control group. Moreover, no differences emerged in other ERP parameters (N1 latency and P3 amplitude and latency) between sessions in both groups. This observation indicates that mental fatigue reduced the attentional resources allocated to the early phase of the discrimination process [36] when the alerting system was engaged, even though the ability to categorize the target for the operational purposes of the task, as indexed by the P3, remained unaltered by fatigue. The alerting network is designed to achieve and maintain an internal state in preparation for perceiving the incoming stimuli and facilitating the subsequent response readiness. Neuroimaging studies have demonstrated that this network is related to an increased activity in the thalamus and the frontoparietal cortical networks, especially in the right hemisphere, and it has been associated with the norepinephrine system arising in the locus coeruleus [8]. This psychophysiological finding, combined with the observation that mental fatigue did not impair the efficiency of the network—but that accuracy was impaired—suggests that the state of alertness during the task, despite having a reduced number of resources available to devote to early discriminative phases, is preserved. This finding has a well-defined behavioral significance: when the alerting system is engaged, even though fatigue depletes attentional resources, they continue to be directed to the system that must facilitate the detection of events that require attention and prompt response, even when the risk of making more errors increases.

On the contrary, regarding the orienting network, fatigue decreased the amplitude of P3, implying the process of stimulus categorization. In contrast, the early phases of stimulus processing were not influenced, as indicated by the stability of the N1 parameters. The orienting network aims to prioritize sensory input by selecting information and shifting the attentional focus from one area or object to another in the visual field. It has been related to the superior parietal lobe, the temporal parietal junction, the frontal eye fields, and the pulvinar, and it has been associated with the acetylcholine system [4,5,6]. Task-relevant dimensions of the stimulus strongly modulate P3 amplitude, which is therefore considered a reliable index of the detection and mostly the categorization of target stimuli; it is the component that indicates the correct categorization of the target stimulus, which is useful for the associated behavioral response and is dependent on the top-down voluntary redirection of attentional resources. In the context of orienting network engagement, its amplitude indexes the number of resources available when spatial attention reorientation is required, and even when fatigue reduces them, these processes are conducted effectively and in adequate timing (P3 latency is in fact not modulated by fatigue). This observation is further supported by the fact that orienting network efficiency was preserved after fatigue, as was the accuracy.

Finally, as regards the executive network, mental fatigue produced a highly selective effect, dependent on whether a congruent or incongruent stimulus was presented; both N2 and P3 amplitudes following the incongruent target alone were significantly reduced by mental fatigue. Testing the difference between congruent and incongruent conditions stresses the response to conflict and response inhibition, factors that primarily modulate the N2 and P3 amplitude in a flanker task [37]. Monitoring and solving conflicts between competing information computed in different neural areas are functions processed by the executive network, linked to the activity of the anterior cingular cortex (ACC) and the lateral prefrontal cortex, whose functioning is influenced by the dopamine system [4,5,6]. N2 represents in fact a psychophysiological signature of the activity of the conflict monitoring system, which relies on top-down resources, especially when the task highlights the processing of conflicting information to select the appropriate response between competing responses [37]. Mental fatigue selectively reduces the resources devoted to these processes and the ones needed to withhold a prepotent motor response, such that response conflict is resolved (P3 amplitude reduction). Furthermore, the fatigue-induced depletion of attentional resources devoted to the incongruent stimulus during recruitment of the executive network caused subjects to pay for maintaining a stable reaction time in terms of reduced accuracy without being able to reduce it, as in the control sample. With time-on-task effects, it has been previously demonstrated that mental fatigue predominantly affects top-down attentional resources rather than bottom-up ones [18]. It is arguable that this effect of mental fatigue on the executive control is related to an impairment in cognitive flexibility, possibly due to an exacerbation of the cognitive cost related to this mental operation. Indeed, task switching is a higher cognitive process, which reallocates cognitive resources during tasks, crucial in everyday life to achieve goal-directed behavior [27], and it has recently been demonstrated that mental fatigue altered the dynamic interactions between different brain regions during task switching [38], enhancing the task-switching cost [39].

In summary, our data indicate that mental fatigue exerts its effects on attentional networks, reducing the amount of resources devoted to the early phase of stimulus processing during alerting activation (N1 amplitude reduction), the categorization process during orienting activation (reduced P3 amplitude), and the response conflict and response inhibition during the executive activation (reduced N2 and P3 amplitudes), while subjects are able to maintain an adequate network efficiency, possibly because their cognitive reserve is still preserved from the effects of aging or even pathological conditions. Indeed, studies conducted on older people have revealed a decline in alerting efficiency with age [40], and also a reduced P3 amplitude for incongruent targets, which was associated with a reduced P3 latency [41], suggesting that healthy aging is associated with a decline in cognitive control. This finding is also confirmed in elderly bilinguals, who are characterized by greater cognitive reserve, i.e., individual differences in susceptibility to age- or disease-related brain changes, but in whom the decline in attentional efficiency is associated with increased activation of fronto-parietal cortices associated with top-down control of attention [42].

This study has some limitations. First, the sample size (15 subjects in each group) was relatively small when compared to studies using behavioral measures such as RT, which may explain why we also found the significance levels and effect sizes to be rather small. Nonetheless, most of the experiments on the effects of fatigue on attention, which have been conducted with ERPS in healthy subjects, have substantially overlapping samples [16,18,21,43,44,45,46]. Second, to induce mental fatigue, we adopted a continuous arithmetic task consisting of a sequence of equations lasting 1 h. The duration of this task was chosen arbitrarily, but the subjects’ ratings of aversion to the task and fatigue were significantly different before and after the sessions in the F group alone. However, these methodological limitations call for caution in generalizing these results.

Mental fatigue represents a burden in many chronic conditions, especially those affecting the nervous system. Studying different patient populations may provide insight into the mechanisms of attentional network disruption induced by this symptom, as well as the potential for rehabilitation through cognitive/working memory training, as demonstrated by studies on ADHD patients [47].

## 5. Conclusions

Our findings support these previous observations, overcoming the potentially confounding factor of ERPs’ habituation. They reveal the covert effects of mental fatigue on attention, indicating that even though healthy young subjects’ compensatory mechanisms possibly allow them to maintain adequate overt performances, fatigue still exerts a negative effect on top-down attentional mechanisms that could potentially be behaviorally relevant, for instance, in pathological populations or elderly people. The use of the ANT in our study was crucial. It provided an ideal framework to evaluate overt performances and effectively disentangled the effects of mental fatigue within the context of selective attention networks.

## Figures and Tables

**Table 1:** Psychological measures prior to and after each ANT session.

	Aversion Scale			POMS—Fatigue		
	Pre	Post	*p* *	Pre	Post	*p* *
NF	0.8 ± 2.3	0.6 ± 1.7	0.64	6.5 ± 3.5	7.1 ± 3.5	0.48
F	0.4 ± 0.7	5.4 ± 2.2	<0.001	9.0 ± 6.3	10.9 ± 6.7	0.02

Values (mean ± SD) depict the subject’s choice in a visual analog scale in which 1 represents poorest aversion and 10 represents maximal aversion to the task and ratings of POMS-fatigue. F and *p* values originate from separate ANOVAs for each measure comparing the group (NF, F) × timing (pre, post). * *p*: after Bonferroni correction. Significance level was set to *p* ≤ 0.05.

**Table 2:** Mean reaction times and accuracy under each condition for fatigue and control groups.

	FLANKER	CUE							
		NC		DC		CC		SC	
Mean RT ms (SD)		PRE	POST	PRE	POST	PRE	POST	PRE	POST
NF	Congruent	570 (63)	516 (51)	532 (49)	492 (59)	532 (57)	489 (58)	493 (68)	444 (65)
	Incongruent	653 (92)	602 (92)	640 (110)	578 (83)	634 (94)	565 (70)	574 (100)	515 (92)
	Neutral	531 (38)	493 (61)	492 (51)	456 (47)	485 (43)	455 (48)	466 (57)	430 (53)
F	Congruent	563 (94)	572 (75)	526 (93)	546 (85)	523 (98)	546 (88)	518 (98)	513 (58)
	Incongruent	655 (138)	662 (124)	609 (120)	636 (129)	592 (123)	625 (133)	593 (118)	588 (104)
	Neutral	516 (106)	560 (78)	487 (92)	500 (56)	488 (79)	506 (61)	500 (84)	504 (60)
% Accuracy (SD)									
NF	Congruent	1.00 (0)	1.00 (0)	1.00 (0)	1.00 (0)	1.00 (0)	1.00 (0)	1.00 (0.02)	1.00 (0)
	Incongruent	0.98 (0.06)	0.98 (0.04)	0.98 (0.03)	0.99 (0.05)	0.99 (0.03)	0.99 (0.03)	1.00 (0.02)	1.00 (0.02)
	Neutral	0.99 (0.02)	1.00 (0)	1.00 (0)	1.00 (0)	0.99 (0.04)	1.00 (0.02)	0.99 (0.02)	1.00 (0)
F	Congruent	1.00 (0.02)	0.99 (0.02)	1.00 (0)	1.00 (0)	1.00 (0)	1.00 (0)	1.00 (0.02)	0.93 (0.26)
	Incongruent	0.99 (0.03)	0.96 (0.06)	0.97 (0.04)	0.97 (0.05)	0.99 (0.03)	0.98 (0.05)	0.99 (0.03)	0.97 (0.04)
	Neutral	1.00 (0)	0.99 (0.02)	1.00 (0)	1.00 (0.02)	1.00 (0.02)	1.00 (0)	1.00 (0.02)	0.99 (0.03)

NC: no cue; DC: double cue; CC: central cue; SC: spatial cue.

## Data Availability

The raw data supporting the conclusions of this article will be made available by the authors on request.
